# Visceral Adipose Tissue and Different Measures of Adiposity in Different Severities of Diffuse Idiopathic Skeletal Hyperostosis

**DOI:** 10.3390/jpm11070663

**Published:** 2021-07-15

**Authors:** Netanja I. Harlianto, Jan Westerink, Wouter Foppen, Marjolein E. Hol, Rianne Wittenberg, Pieternella H. van der Veen, Bram van Ginneken, Jonneke S. Kuperus, Jorrit-Jan Verlaan, Pim A. de Jong, Firdaus A. A. Mohamed Hoesein, on behalf of the UCC-SMART-Study Group

**Affiliations:** 1 Department of Radiology, University Medical Center Utrecht and Utrecht University, 3584 CX Utrecht, The Netherlands; W.Foppen@umcutrecht.nl (W.F.); M.E.Hol-6@umcutrecht.nl (M.E.H.); erika_vanderveen@hotmail.com (P.H.v.d.V.); P.deJong-8@umcutrecht.nl (P.A.d.J.); F.A.A.MohamedHoesein@umcutrecht.nl (F.A.A.M.H.); 2 Department of Orthopedic Surgery, University Medical Center Utrecht and Utrecht University, 3584 CX Utrecht, The Netherlands; J.S.Kuperus@umcutrecht.nl (J.S.K.); J.J.Verlaan@umcutrecht.nl (J.-J.V.); 3 Department of Vascular Medicine, University Medical Center Utrecht and Utrecht University, 3584 CX Utrecht, The Netherlands; J.Westerink-3@umcutrecht.nl; 4 Department of Radiology, Netherlands Cancer Institute, 1066 CX Amsterdam, The Netherlands; Rianne_wittenberg@hotmail.com; 5 Department of Medical Imaging, Radboud University Medical Center, 6525 GA Nijmegen, The Netherlands; bram.vanginneken@radboudumc.nl

**Keywords:** diffuse idiopathic skeletal hyperostosis, risk factors, adiposity, intra-abdominal fat

## Abstract

Background: Diffuse idiopathic skeletal hyperostosis (DISH) is associated with both obesity and type 2 diabetes. Our objective was to investigate the relation between DISH and visceral adipose tissue (VAT) in particular, as this would support a causal role of insulin resistance and low grade inflammation in the development of DISH. Methods: In 4334 patients with manifest vascular disease, the relation between different adiposity measures and the presence of DISH was compared using *z*-scores via standard deviation logistic regression analyses. Analyses were stratified by sex and adjusted for age, systolic blood pressure, diabetes, non-HDL cholesterol, smoking status, and renal function. Results: DISH was present in 391 (9%) subjects. The presence of DISH was associated with markers of adiposity and had a strong relation with VAT in males (OR: 1.35; 95%CI: 1.20–1.54) and females (OR: 1.43; 95%CI: 1.06–1.93). In males with the most severe DISH (extensive ossification of seven or more vertebral bodies) the association between DISH and VAT was stronger (OR: 1.61; 95%CI: 1.31–1.98), while increased subcutaneous fat was negatively associated with DISH (OR: 0.65; 95%CI: 0.49–0.95). In females, increased subcutaneous fat was associated with the presence of DISH (OR: 1.43; 95%CI: 1.14–1.80). Conclusion: Markers of adiposity, including VAT, are strongly associated with the presence of DISH. Subcutaneous adipose tissue thickness was negatively associated with more severe cases of DISH in males, while in females, increased subcutaneous adipose tissue was associated with the presence of DISH.

## 1. Introduction

Diffuse idiopathic skeletal hyperostosis (DISH) is a common condition characterized by abnormal hyperostosis with the formation of new bony bridges around ligaments, tendons, and joint capsules. DISH is most frequently present near the anterior longitudinal ligament of the spine but can also manifest in the peripheral skeleton [1]. The exact pathophysiology of DISH remains unclear, but various genetic, metabolic, and inflammatory pathways are likely involved [1]. DISH is more prevalent in older individuals, mostly affects males, and has been associated with several metabolic factors including obesity, hypertension, type 2 diabetes mellitus, and the metabolic syndrome [1,2,3,4]. Furthermore, individuals with DISH are more prone to spinal fractures and cardiovascular events such as stroke [1]. While usually asymptomatic, reported symptoms related to DISH include dysphagia, airway obstruction, and a reduced range of motion [1].

Abdominal obesity, also referred to as central or visceral obesity, is characterized by an increased volume of visceral adipose tissue (VAT) surrounding the intra-abdominal organs [5]. Abdominal obesity is an independent risk factor for cardiovascular disease, and has been related to different pathologies, including dyslipidemia, insulin resistance, cardiovascular disease, diabetes, and cancer [6,7]. VAT is known to produce various inflammatory cytokines and adipokines, the latter contributing to the development of insulin resistance in patients with increased depositions of VAT [8].

Different methods exist to quantify abdominal obesity. General obesity is most commonly quantified using body mass index (BMI), but BMI is limited in differentiating between lean and fat body mass. More accurate approximations of VAT include indirect anthropometric measurements with the waist circumference and waist-to-hip ratio, or direct measurements with ultrasonography or computed tomography (CT) imaging [9].

Previous studies have shown that obesity is associated with both DISH and cardiovascular disease [1,3], however, it is unknown how obesity leads to a higher prevalence of DISH. Moreover, the adiposity measurement showing the strongest relation with DISH is also not known. A strong relation between markers of adiposity with the closest approximation of visceral adiposity may suggest a causal role of insulin resistance and low grade inflammation in the pathogenesis of DISH. Therefore, in the present study we aimed to investigate the relation between DISH and different measurements of adiposity, including VAT. The secondary aim was to compare the relation between adiposity measurements and different severities of DISH, to analyze how the extent of ossification relates to each measure of adiposity.

## 2. Materials and Methods

### 2.1. Study Population

**Figure 1:**
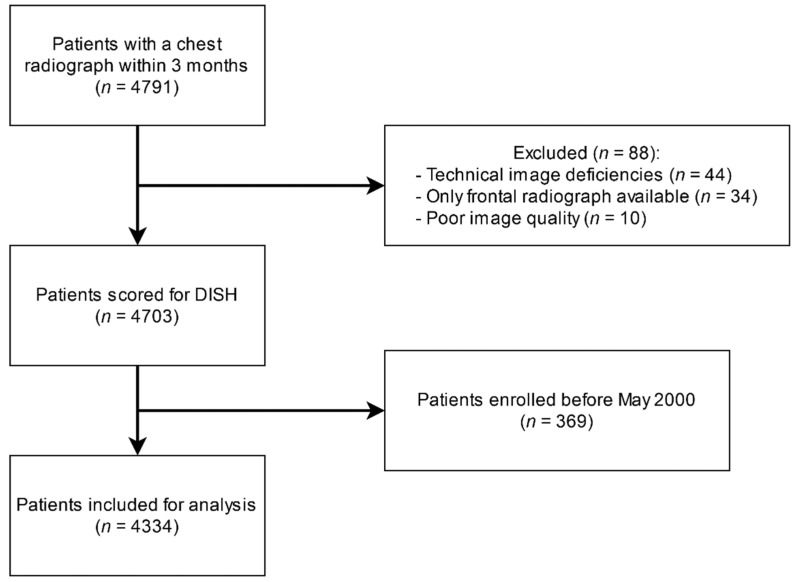
Flow chart of patient selection.

Patients enrolled in the Second Manifestations of ARTerial disease (UCC-SMART) study, an ongoing prospective cohort study of the University Medical Center Utrecht, with a patient population between 18 and 79 years with either manifest or risk factors for vascular disease were included. All patients provided written informed consent at inclusion. The UCC-SMART study is in accordance with the declaration of Helsinki and has been approved by our institutional review board (NL45885.041.13). Patients underwent extensive vascular screening: patients were asked to complete a health questionnaire covering medical history, risk factors, smoking and drinking habits, and prescribed drugs. A standardized diagnostic protocol was followed consisting of a physical examination and laboratory testing in a fasting state. A more detailed description of the UCC-SMART study protocol has been published previously [10]. We identified all patients from the UCC-SMART cohort who received a digital chest radiograph within three months of inclusion, resulting in 4791 available patients. Of this population, 88 patients were subsequently excluded due to technical image deficiencies (*n* = 44), only the frontal radiograph being available (*n* = 34), and poor image quality (*n* = 10). For the current study, we also excluded patients enrolled before May 2000 as visceral fat measurements were not regularly performed before that date. In the end, 4334 patients were available for inclusion (Figure 1).

### 2.2. Assessment of DISH

Using the Resnick criteria [11], chest radiographs were assessed for the presence of DISH. These classification criteria include the presence of ossification of at least four contiguous vertebrae, (relative) preservation of the intervertebral disc height, and the absence of apophyseal joint bony ankylosis or sacroiliac joint erosion. The chest radiographs were scored by a group of six readers from the department of Radiology of our institution, all of whom were certified to read chest radiographs independently (entrusted professional activity level 4 or 5). To analyze the extent of anterolateral ossification in relation to the markers of adiposity, the severity of DISH was also scored depending on the number of involved vertebral bodies with adjacent bony bridges. Although no standardized criteria have been validated for scoring different severities of DISH, we classified the severity of DISH as the following: grade 1 DISH indicated flowing bridging osteophytes of 4 adjacent vertebral bodies; grade 2 DISH indicated flowing bridging osteophytes of 5 or 6 vertebral bodies; and grade 3 DISH indicated flowing bridging osteophytes of 7 or more vertebral bodies.

### 2.3. Measurements of Adiposity Markers

Body mass index (BMI) was calculated by dividing the weight by the squared height (kg/m^2^). Waist circumference was measured halfway between the lower rib and the iliac crest in the standing position. Hip circumference was measured at the level of the greater trochanter in the standing position. The waist-to-hip ratio was calculated using the waist circumference divided by the hip circumference. To measure subcutaneous and intra-abdominal fat, B-mode ultrasound of the abdomen was obtained and performed by well-trained registered vascular technologists in a certified vascular laboratory. Measurements were made with the patient in supine position using an ATL HDI 3000 (Philips Medical Systems, Eindhoven, The Netherlands) with a C4-2 transducer without prior bowel preparation. Good reproducible results (interobserver coefficient of variation of 5.4%) and a strong association (Pearson’s correlation coefficient of 0.81, *p* < 0.001) were found when comparing ultrasonographic measurements with a subset of CT scans for intra-abdominal fat in our cohort [12]. Adhering to a strict protocol, measurements were performed using electronic calipers at the end of a quiet inspiration, applying minimal pressure without displacement or compression of the abdominal cavity. The transducer was placed in a straight line drawn between the left and right midpoints of the lower rib and the iliac crest. Three different measurements at three different positions were performed. Subcutaneous fat was measured as the distance between the linea alba and the skin. Intra-abdominal fat was measured as the distance between the peritoneum and the lumbar spine or psoas muscles. The contribution of VAT to total abdominal fat (VAT%) was calculated as [100 × VAT ÷ (VAT + SAT)] to evaluate the impact of an increased VAT, independent of other adipose tissue locations or height.

### 2.4. Statistics

Categorical variables were expressed using frequencies and percentages, and normally distributed continuous variables using the mean and standard deviation. Positively skewed data were transformed using logarithmic transformation. Univariate and multivariate logistic regression analyses were performed for each of the population characteristics with the presence or absence of DISH as outcome, adjusted for age and sex. Risk was calculated using odds ratios (OR) with 95% confidence intervals (95% CI). Specifically for the adiposity measurements, data were transformed using z-scores for a per standard deviation (SD) analysis using a stepwise adjusted approach including confounder selection based upon literature and etiologic considerations with sex-stratification. In addition to the crude analysis, two models were used: model two was adjusted for age, and model three additionally adjusted for cardiovascular risk factors such as renal function calculated with the Chronic Kidney Disease Epidemiology Collaboration equation (CKD-EPI) [13], systolic blood pressure, diabetes, smoking status, and non-high density lipoprotein (HDL) cholesterol. Missing data (1%) were imputed using multiple imputation based on the Markov Chain Monte Carlo method (*n* = 10 and 40 iterations) and estimates for statistical inference were pooled according to Rubin’s Rules [14]. Significance was set at *p* < 0.05. Data analysis was performed using R, version 3.6.3 (R Foundation for Statistical Computing, Vienna, Austria) using the mice package [15].

## 3. Results

### 3.1. Baseline Characteristics

A total of 4334 patients were included, of whom 391 (9.0%) satisfied the criteria for DISH. A total of 146 patients were classified as grade 1 DISH, 131 as grade 2 DISH, and 114 as grade 3 DISH. Population characteristics are summarized in Table 1. Compared to patients without DISH, subjects with DISH were older (67 vs. 59 years) and more often male (85.7% vs. 68.4%). Furthermore, DISH subjects had significantly more metabolic syndrome (66% vs. 52%) and diabetes (30% vs. 20%). All adiposity measurements except subcutaneous fat were increased in patients with DISH when compared with patients without DISH.

### 3.2. Risk Factors for DISH

Results of logistic regression analyses are listed in Table 2. After adjusting for age and sex, DISH was significantly associated with presence of metabolic syndrome (OR 1.78 (95%CI: 1.43–2.24)), the presence of diabetes (OR 1.50 (95%CI: 1.18–1.91)), and glucose (per 1 mmol/L) (OR 1.10 (95%CI: 1.04–1.17)). Systolic blood pressure (per 1 mmHg), the presence of hypertension, and pulse pressure (per 1 mmHg) were also associated with DISH, whereas diastolic blood pressure was not. Regarding blood lipid profile, DISH was associated with HDL-cholesterol.

### 3.3. Intra-Abdominal Fat Measurements and Adiposity Markers in Relation to DISH in Males

Results of adiposity measurements with an increase of 1 SD in relation to the presence of DISH in males are listed in Table 3. In the crude analysis, the presence of DISH was associated with the adiposity measures weight, BMI, waist circumference, subcutaneous fat, VAT, and VAT%. After full adjustments, the significant adiposity markers were weight (OR 1.56; 95%CI: 1.36–1.79), BMI (OR 1.58; 95%CI: 1.28–1.94), waist circumference (OR 1.45; 95%CI: 1.15–1.82), and VAT (OR 1.35; 95%CI: 1.20–1.54). An increase of 1 SD of subcutaneous fat, the waist-to-hip ratio, or VAT% was not significantly associated with the presence of DISH. In general, the adiposity measures weight, BMI, waist circumference, and VAT were significant for all grades of DISH in crude and full adjusted analyses. In the most severe DISH group, the relation between VAT and the presence of DISH became stronger (OR 1.61; 95%CI: 1.31–1.98). Moreover, in this group with most severe DISH, 1 SD increase in subcutaneous fat was negatively associated with the presence of DISH (OR 0.65; 95%CI: 0.49–0.95), whereas VAT% was positively associated with the presence of DISH (OR 1.80; 95%CI: 1.25–2.68). These relations for subcutaneous fat and VAT% were not observed in the groups with grade 1 or grade 2 DISH.

### 3.4. Intra-Abdominal Fat Measurements and Adiposity Markers in Relation to DISH in Females

Table 4 lists the results of adiposity measures in females in relation to the presence of DISH. The presence of DISH was related to the markers weight (OR 1.52; 95%CI: 1.20–1.94), BMI (OR 1.55; 95%CI: 1.28–1.89), waist circumference (OR 1.54; 95%CI: 1.06–2.24), and VAT (OR 1.71; 95%CI: 1.33–2.19). After adjusting for cardiovascular risk factors, the relation between the presence of DISH and waist circumference became attenuated (OR 1.39; 95%CI: 0.89–2.16), while an increase by 1 SD of subcutaneous fat was associated with the presence of DISH (OR 1.43; 95%CI: 1.14–1.80). The adiposity markers weight (OR 1.75; 95%CI: 1.29–2.38), BMI (OR 1.66; 95%CI: 1.30–2.13), and VAT (OR 1.43; 95%CI: 1.06–1.93) remained significantly associated after full adjustment. For the different Grades of DISH, the adiposity measures weight and BMI were significant for all grades of DISH in crude and full adjusted analyses.

## 4. Discussion

In the current study, we aimed to assess the relation between different severities of DISH and various measurements of adiposity in both males and females with a high risk for cardiovascular disease. We found that, in males, all adiposity markers except for subcutaneous fat and the waist-to-hip ratio were associated with the presence of DISH. When analyzing the group with the most severe DISH, the relation between VAT and the presence of DISH became stronger. Moreover, increased subcutaneous fat was negatively associated with cases of DISH with extensive ossification, reinforcing the importance of adipose tissue distribution in the pathogenesis of DISH.

In females, the adiposity markers we identified with the presence of DISH were weight, BMI, subcutaneous fat, and VAT. Waist circumference was not associated with the presence of DISH, which was the case for males, whereas in female DISH patients increased subcutaneous fat was positively associated with the presence of DISH.

The risk factors we identified for DISH in our cohort also strongly relate to the presence of VAT and obesity [16] showing the probable causal relation between VAT and insulin resistance. The formation of bone in DISH is potentially linked with metabolic derangements via the insulin-like growth factor-I pathway, which is able to induce proliferation in chondrocytes and osteoblasts [17].

The prevalence of DISH in our cohort was 9.0% and our data confirm previously observed associations between DISH and BMI [3,18,19,20,21], diabetes [3,19,20,21], waist circumference [5,18,22], metabolic syndrome [5,18], systolic blood pressure [18,23], and hypertension [5,18]. A higher level of HDL-cholesterol was significantly associated with the presence of DISH in our study, whereas other cohorts did not find this relation [5,18]. These risk factors are described to strongly relate to excess levels of VAT and the presence of insulin resistance [16]. In line with previous work, no association was found between DISH and hsCRP [18]. As our patient population had increased risk for cardiovascular disease, a large portion of our cohort was treated with statin therapy for cardiovascular risk management. The use of statins is associated with a reduction in levels of hsCRP [24], which may explain why no significant difference was observed for hsCRP between the groups with and without DISH in our cohort.

Our results show that the presence of DISH is associated with VAT, which is in accordance with Lantsman et al. [25] and Okada et al. [26], who measured VAT in DISH patients using CT imaging. In the study by Okada and colleagues, the area of VAT was significantly increased in DISH patients (130.7 ± SD 58.2 cm^2^ vs. 89.0 ± SD 48.1 cm^2^).

Interestingly, females with DISH had both increased subcutaneous fat and VAT in our cohort. Contrarily in males, an increased VAT was linked with DISH while increased subcutaneous fat was not. When estimating the percentage of VAT in relation to total abdominal fat, no association was found between VAT% and DISH for both sexes. This might be explained by the poor reliability of using adiposity measurements with ultrasound as proxies for VAT accumulation in relation to total abdominal fat. Ideally, CT-based segmentations in the coronal plane are preferred as this can more accurately measure the total area of visceral fat in relation to the total area of abdominal fat. To minimize this discrepancy, our measurements adhered to a strict protocol, and the estimations were averaged over multiple measurements of the same patient.

Although other adiposity markers had stronger observed associations with DISH compared to VAT in our study, our results still indicate that one SD increase of VAT is associated with a 35% and 43% increase in risk for DISH in males and females, respectively. VAT is known to increase with older age, and a higher percentage of VAT is found in men [27,28]. Furthermore, it is now well established that VAT produces different adipokines and inflammatory molecules including leptin, adiponectin, tumor necrosis factor-α, and interleukin-6. In the literature, few studies have reported these adipokines in relation to DISH. Visceral obesity results in lower levels of adiponectin [29], which was reported for DISH in two studies [30,31]. Moreover, increased levels of leptin [31,32] and visfatin [30] were also observed in DISH patients. Both leptin and adiponectin are known to influence bone metabolism and bone homeostasis [31,33]. An adequate explanation for the role of these adipokines in the pathogenesis of DISH remains to be determined. Recently, Mader et al. [34] reviewed the involvement of a possible inflammatory component in DISH, and concluded that local inflammation, prior to or as a consequence of metabolic derangements, could play a crucial role in the development of DISH. Our results support the notion that research on VAT and inflammation should be further (re)explored in patients with DISH.

### Strengths and Limitations

The strengths of our study are the relatively large sample size of our prospective cohort, with extensive and accurate information on a broad array of cardiovascular risk factors. Moreover, we studied the relative importance of adiposity measurements and corrected for confounders, which has not been reported previously in DISH.

Our study, however, also has limitations. Visceral and subcutaneous fat measured with ultrasonography have been reported to be prone to measurement variability. However, an interobserver coefficient of variation of 5.4% was found for our cohort, indicating good measurement reliability [12]. Secondly, the Resnick criteria for DISH are arbitrary and some milder forms or earlier stages of DISH will be misclassified. This can result in some underestimation of the associations. Finally, the cross-sectional design of our study should warrant a cautious approach when drawing causal etiological conclusions.

## 5. Conclusions

To summarize, measurements of adiposity, including visceral adipose tissue thickness, were associated with the presence of DISH in both males and females. Subcutaneous adipose tissue thickness was negatively associated in males with most severe DISH. In females, subcutaneous adipose tissue was positively associated with the presence of DISH. Our research supports further investigation into the role of visceral adipose tissue and insulin resistance in the pathogenesis of DISH.

## Figures and Tables

**Table 1:** Baseline patient characteristics.

Variable	Total Group (*n* = 4334)	No DISH (*n* = 3943)	Total DISH (*n* = 391)	Grade 1 DISH (*n* = 146)	Grade 2 DISH (*n* = 131)	Grade 3 DISH (*n* = 114)
Age (years), mean (SD)	58.5 (±11.3)	57.8 (±11.3)	66.1 (±7.7)	65.2 (±8)	65.6 (±7.6)	68 (±7)
Sex (male), %	70.0%	68.4%	85.7%	80.1%	87.8%	83%
Diabetes, %	19.3%	18.3%	29.4%	25.3%	32.1%	31.6%
Glucose (mmol/L), mean (SD)	6.3 (±1.7)	6.3 (±1.7)	6.6 (±1.5)	6.5 (±1.4)	6.8 (±1.6)	6.6 (±1.3)
HbA1c (%), mean (SD)	5.9 (±0.9)	5.9 (±1)	6 (±0.8)	5.9 (±0.7)	6.1 (±0.9)	5.8 (±1)
CKD EPI (mL/min/1.73 m^2^), mean (SD)	78.5 (±19)	79.1 (±19.1)	73 (±17.5)	73.4 (±17.9)	74.1 (±17.3)	71.3 (±17.2)
Systolic blood pressure (mmHg), mean (SD)	140.9 (±21.7)	140.5 (±21.6)	145.8 (±22.2)	144.8 (±22.8)	143.4 (±21.1)	149.9 (±22)
Diastolic blood pressure (mmHg), mean (SD)	83.2 (±12.7)	83.3 (±12.8)	82.4 (±12.1)	83.1 (±12.7)	80.7 (±11.4)	83.5 (±11.9)
Hypertension, % ^#^	24.6%	24.1%	30.2%	29%	23.1%	40.3%
Pulse pressure (mmHg), mean (SD)	57.8 (±15.3)	57.2 (±15.2)	63.4 (±16)	61.8 (±15.3)	62.6 (±16.6)	66.4 (±15.7)
HDL-cholesterol (mmol/L), mean (SD)	1.3 (±0.4)	1.3 (±0.4)	1.2 (±0.3)	1.2 (±0.3)	1.2 (±0.4)	1.2 (±0.3)
LDL-cholesterol (mmol/L), mean (SD)	2.8 (±1.1)	2.8 (±1)	2.7 (±1.1)	2.8 (±1.2)	2.7 (±0.9)	2.6 (±1)
Triglycerides (mmol/L), mean (SD) ^&^	0.94 (±0.37)	0.92 (±0.36)	0.93 (±0.34)	0.96 (±0.46)	0.93 (±0.31)	0.98 (±0.32)
Non-HDL cholesterol (mmol/L), mean (SD)	3.6 (±1.2)	3.6 (±1.2)	3.5 (±1.3)	3.6 (±1.5)	3.4 (±1.2)	3.4 (±1.1)
hsCRP (mg/L), mean (SD) ^&^	1.24 (±0.78)	1.23 (±0.78)	1.29 (±0.77)	1.25 (±0.72)	1.26 (±0.79)	1.36 (±0.80)
Metabolic syndrome, % ^#^	53.3%	52.2%	65.9%	63.7%	63.3%	68.4%
Smoking (current vs. former), % ^#^	72.7%	72.3%	77.0%	75.1%	78.5%	79.6%
Packyears, mean (SD)	17.3 (±19.5)	17.2 (±19.4)	18.6 (±20.2)	18 (±20.5)	19 (±19.5)	19 (±20.6)
Drinking (current vs. former), % ^#^	80.9%	80.4%	86.2%	83.4%	89.2%	87.7%
History of cerebral vascular disease, % ^#^	15.7%	15.2%	14.1%	15.1%	10.7%	16.7%
History of coronary artery disease (%) ^#^	50.5%	49.5%	59.8%	56.2%	64.1%	59.6%
History of peripheral artery disease, % ^#^	9.1%	9.2%	7.9%	8.9%	7.6%	7%
History of abdominal aortic aneurysm, % ^#^	5.3%	4.9%	8.7%	8.2%	8.4%	9.6%
Weight (kg), mean (SD)	82.7 (±15.8)	82.1 (±15.8)	88.2 (±15.4)	87.5 (±15.6)	87.8 (±14.7)	89.4 (±15.9)
BMI (kg/m^2^), mean (SD)	27.1 (±4.5)	26.9 (±4.4)	27.8 (±4.5)	28.8 (±4.5)	28.5 (±4.7)	29 (±4.4)
Waist circumference (cm), mean (SD)	95.5 (±13.1)	94.9 (±8.7)	102 (±12.2)	101.1 (±12.7)	101.5 (±11.2)	103.6 (±12.7)
Waist- to-hip ratio, mean (SD)	0.92 (±0.09)	0.91 (±0.09)	0.96 (±0.07)	0.94 (±0.08)	0.95 (±0.06)	0.97 (±0.08)
Subcutaneous fat (cm), mean (SD)	2.4 (±1.2)	2.4 (±1.2)	2.1 (±1.3)	2.3 (±1.5)	2.2 (±1.1)	1.8 (±0.9)
Visceral fat (cm), mean (SD)	9 (±2.7)	8.9 (±2.6)	10.1 (±2.8)	9.8 (±2.8)	9.9 (±2.7)	10.7 (±2.9)

^#^ Percentages were calculated after excluding missing cases from the denominator; ^&^ Log-transformed; Data are displayed using number (percentage) for categorical variables and mean (±standard deviation) for normally continuous data. BMI: body mass index; hsCRP: high sensitivity c-reactive protein; CKD-EPI: Chronic Kidney Disease Epidemiology Collaboration; HDL: high density lipoprotein; LDL: low density lipoprotein.

**Table 2:** Risk factor analysis for the DISH group.

Variable	Units	Univariate Model		Age + Sex Adjusted	
		OR (95%CI)	*p*-Value	OR (95%CI)	*p*-Value
Age *	+1 year	1.09 (1.08–1.10)	<0.001	1.09 (1.08–1.11)	<0.001
Sex ^#^	Male vs. female	2.78 (2.08–3.7)	<0.001	2.86 (2.13–3.85)	<0.001
Diabetes	Present vs. absent	1.72 (1.36–2.16)	<0.001	1.50 (1.18–1.91)	<0.001
Glucose	+1 mmol/L	1.1 (1.05–1.16)	<0.001	1.1 (1.04–1.17)	<0.001
HbA1c	+1%	1.14 (1.03–1.27)	0.01	1.13 (0.99–1.27)	0.06
CKD-EPI	+1 mL/min/1.73 m^2^	0.98 (0.98–0.99)	<0.001	1.0 (0.99–1.01)	0.20
Systolic blood pressure	+1 mmHg	1.01 (1.00–1.02)	<0.001	1.01 (1.00–1.01)	0.008
Diastolic blood pressure	+1 mmHg	0.99 (0.99–1.00)	0.21	1.00 (0.99–1.01)	0.55
Hypertension	Present vs. absent	1.36 (1.09–1.72)	0.007	1.43 (1.13–1.82)	0.003
Pulse pressure	+1 mmHg	1.02 (1.02–1.03)	<0.001	1.01 (1.00–1.02)	0.001
HDL-cholesterol	+1 mmol/L	0.66 (0.49–0.88)	0.005	0.68 (0.49–0.94)	0.02
LDL-cholesterol	+1 mmol/L	0.92 (0.83–1.01)	0.08	1.05 (0.93–1.18)	0.43
Triglycerides ^&^	+1 log(1 mmol/L)	1.05 (0.88–1.27)	0.58	1.33 (1.08–1.63)	0.006
Non HDL-cholesterol	+1 mmol/L	0.94 (0.86–1.02)	0.14	1.11 (1.01–1.22)	0.03
hsCRP ^&^	+1 log(1 (mg/L)	1.07 (0.97–1.19)	0.18	1.05 (0.95–1.17)	0.30
Metabolic syndrome	Present vs. absent	1.69 (1.36–2.11)	<0.001	1.78 (1.43–2.24)	<0.001
Smoking	Current vs. former	1.31 (1.02–1.68)	0.03	1.03 (0.79–1.34)	0.82
Packyears	+1 packyear	1.00 (0.99–1.01)	0.15	1.00 (0.99–1.00)	0.38
Drinking	Current vs. former drinker	1.54 (1.14–2.09)	0.004	1.12 (0.81–1.54)	0.51
History of cerebral vascular disease	Yes vs. no	0.92 (0.67–1.22)	0.56	0.79 (0.57–1.06)	0.13
History of coronary artery disease	Yes vs. no	1.52 (1.23–1.88)	<0.001	0.91 (0.72–1.14)	0.39
History of peripheral artery disease	Yes vs. no	0.85 (0.57–1.22)	0.4	0.74 (0.49–1.08)	0.13
History of abdominal aortic aneurysm	Yes vs. no	1.84 (1.24–2.66)	0.002	1.02 (0.67–1.49)	0.94

* Sex adjusted; ^#^ Age adjusted; ^&^ Log-transformed. OR: odds ratio; CI: confidence interval; BMI: body mass index; hsCRP: high sensitivity c-reactive protein; CKD-EPI: Chronic Kidney Disease Epidemiology Collaboration; HDL: high density lipoprotein; LDL: low density lipoprotein.

**Table 3:** Adiposity measurements per SD with different severities of DISH as outcome in males.

	Model	Total DISH	Grade 1 DISH	Grade 2 DISH	Grade 3 DISH
		OR (95%CI)	OR (95%CI)	OR (95%CI)	OR (95%CI)
Weight (kg), per SD increase	1	1.24 (1.10–1.39) ^a^	1.26 (1.05–1.51) ^a^	1.16 (0.96–1.41) ^a^	1.30 (1.07–1.58) ^a^
	2	1.59 (1.39–1.81) ^a^	1.53 (1.26–1.87) ^a^	1.44 (1.17–1.77) ^a^	1.81 (1.45–2.25) ^a^
	3	1.56 (1.36–1.79) ^a^	1.54 (1.26–1.89) ^a^	1.40 (1.14–1.74) ^a^	1.73 (1.39–2.17) ^a^
BMI (kg/m^2^), per SD increase	1	1.39 (1.20–1.60) ^a^	1.38 (1.12–1.70) ^a^	1.27 (1.05–1.54) ^a^	1.44 (1.14–1.83) ^a^
	2	1.60 (1.31–1.94) ^a^	1.51 (1.14–2.00) ^a^	1.41 (1.10–1.79) ^a^	1.71 (1.17–2.51) ^a^
	3	1.58 (1.28–1.94) ^a^	1.53 (1.13–2.09) *	1.38 (1.08–1.77) ^a^	1.66 (1.16–2.39) ^a^
Waist circumference (cm), per SD increase	1	1.44 (1.20–1.71) ^a^	1.41 (1.16–1.73) ^a^	1.33 (1.07–1.66) ^a^	1.53 (1.15–2.04) ^a^
	2	1.47 (1.18–1.83) ^a^	1.43 (1.14–1.79) ^a^	1.35 (1.05–1.75) ^a^	1.59 (1.10–2.29) ^a^
	3	1.45 (1.15–1.82) ^a^	1.44 (1.13–1.83) ^a^	1.32 (1.01–1.72) ^a^	1.53 (1.07–2.18) ^a^
Waist-to-hip ratio, per SD increase	1	1.40 (0.97–2.01)	1.37 (1.02–1.84) ^a^	1.27 (0.90–1.79)	1.54 (0.89–2.66)
	2	1.32 (0.94–1.87)	1.30 (0.98–1.74)	1.20 (0.86–1.69)	1.48 (0.86–2.53)
	3	1.29 (0.92–1.82)	1.30 (0.97–1.76)	1.16 (0.83–1.63)	1.42 (0.85–2.36)
Subcutaneous fat (cm), per SD increase	1	0.81 (0.68–0.95) ^a^	0.90 (0.71–1.14)	0.96 (0.76–1.21)	0.53 (0.37–0.76) ^a^
	2	0.95 (0.81–1.10)	1.02 (0.81–1.29)	1.10 (0.87–1.38)	0.64 (0.44–0.94) ^a^
	3	0.95 (0.82–1.11)	1.02 (0.81–1.28)	1.10 (0.88–1.37)	0.65 (0.49–0.95) ^a^
VAT (cm), per SD increase	1	1.37 (1.22–1.54) ^a^	1.30 (1.08–1.56) ^a^	1.24 (1.03–1.51) ^a^	1.64 (1.35–1.97) ^a^
	2	1.38 (1.22–1.56) ^a^	1.29 (1.07–1.57) ^a^	0.24 (1.02–1.51) ^a^	1.68 (1.38–2.05) ^a^
	3	1.35 (1.20–1.54) ^a^	1.30 (1.06–1.59) ^a^	1.21 (0.98–1.49)	1.61 (1.31–1.98) ^a^
VAT%, per SD increase	1	1.39 (1.18–1.65)	1.25 (0.99–1.59)	1.10 (0.87–1.40)	2.19 (1.55–3.10) ^a^
	2	1.21 (1.02–1.43) ^a^	1.11 (0.87–1.42)	0.97 (0.76–1.23)	1.87 (1.30–2.66) ^a^
	3	1.18 (0.99–1.39)	1.10 (0.86–1.41)	0.94 (0.74–1.20)	1.80 (1.25–2.68) ^a^

Model 1: DISH crude; Model 2: adjusted for age; Model 3: adjusted for age, systolic blood pressure, diabetes, non-HDL cholesterol, smoking status, and renal function. ^a^ *p* < 0.05, SD: standard deviation; OR: odds ratio; CI: confidence interval; BMI: body mass index; VAT: visceral adipose tissue; VAT%: visceral adipose tissue in relation to total abdominal fat.

**Table 4:** Adiposity measurements per SD with different severities DISH as outcome in females.

	Model	Total DISH	Grade 1 DISH	Grade 2 DISH	Grade 3 DISH
		OR (95%CI)	OR (95%CI)	OR (95%CI)	OR (95%CI)
Weight (kg), per SD increase	1	1.52 (1.20–1.94) ^a^	1.36 (0.97–1.91)	1.71 (1.15–2.54) ^a^	1.57 (0.96–2.58)
	2	1.94 (1.46–2.57) ^a^	1.68 (1.14–2.47) ^a^	2.20 (1.39–3.49) ^a^	2.21 (1.20–4.04) ^a^
	3	1.75 (1.29–2.38) ^a^	1.59 (1.04–2.43) ^a^	1.84 (1.11–3.04) ^a^	2.08 (1.08–4.03) ^a^
BMI (kg/m^2^), per SD increase	1	1.55 (1.28–1.89) ^a^	1.42 (1.09–1.84) ^a^	1.66 (1.20–2.29) ^a^	1.57 (1.08–2.30) ^a^
	2	1.13 (1.08–1.20) ^a^	1.60 (1.18–2.16) ^a^	1.93 (1.34–2.80) ^a^	1.97 (1.24–3.17) ^a^
	3	1.66 (1.30–2.13) ^a^	1.55 (1.11–2.16) ^a^	1.72 (1.16–2.55) ^a^	1.89 (1.12–3.17) ^a^
Waist circumference (cm), per SD increase	1	1.54 (1.06–2.24) ^a^	1.37 (0.89–2.10)	1.69 (1.10–2.59) ^a^	1.68 (0.91–3.11)
	2	1.54 (0.99–2.38)	1.33 (0.82–2.18)	1.69 (1.05–2.72) ^a^	1.69 (0.80–3.61)
	3	1.39 (0.89–2.16)	1.24 (0.71–2.18)	1.44 (0.89–2.31)	1.62 (0.74–3.53)
Waist-to-hip ratio, per SD increase	1	1.31 (0.83–2.06)	1.05 (0.64–1.73)	1.44 (0.87–2.40)	1.57 (0.72–3.49)
	2	1.15 (0.76–1.75)	0.86 (0.47–1.57)	1.33 (0.81–2.18)	1.47 (0.64–3.39)
	3	1.03 (0.65–1.64)	0.77 (0.38–1.60)	1.15 (0.67–1.99)	1.48 (0.60–3.60)
Subcutaneous fat (cm), per SD increase	1	1.21 (0.97–1.52)	1.34 (1.04–1.74) ^a^	1.21 (0.72–2.04)	0.81 (0.44–1.48)
	2	1.44 (1.15–1.81) ^a^	1.58 (1.20–2.10) ^a^	1.39 (0.85–2.29)	0.94 (0.49–1.81)
	3	1.43 (1.14–1.80) ^a^	1.55 (1.16–2.08) ^a^	1.48 (0.90–2.44)	0.93 (0.47–1.82)
VAT (cm), per SD increase	1	1.71 (1.33–2.19) ^a^	1.47 (1.04–2.05)	2.08 (1.35–3.19) ^a^	1.72 (1.02–2.88) ^a^
	2	1.63 (1.24–2.13) ^a^	1.36 (0.94–1.98)	2.05 (1.31–3.22) ^a^	1.66 (0.93–2.97)
	3	1.43 (1.06–1.93) ^a^	1.26 (0.84–1.92)	1.61 (0.97–2.65)	1.46 (0.78–2.75)
VAT%, per SD increase	1	1.21 (0.91–1.61)	0.98 (0.69–1.40)	2.07 (1.35–3.19) ^a^	1.75 (0.94–3.26)
	2	1.10 (1.06–1.13) ^a^	0.80 (0.55–1.17)	1.19 (0.61–2.35)	1..46 (0.75–2.83)
	3	0.90 (0.67–1.22)	0.76 (0.51–1.14)	0.97 (0.50–1.89)	1.36 (0.69–2.72)

Model 1: DISH crude; Model 2: adjusted for age; Model 3: adjusted for age, systolic blood pressure, diabetes, non-HDL cholesterol, smoking status, and renal function. ^a^ *p* < 0.05, SD: standard deviation; OR: odds ratio; CI: confidence interval; BMI: body mass index; VAT: visceral adipose tissue; VAT%: visceral adipose tissue in relation to total abdominal fat.

## Data Availability

The informed consent that was signed by the study participants is not compliant with publishing individual data in an open access institutional repository or as supporting information files with the published paper. However, a data request can be sent to the SMART Steering Committee at uccdatarequest@umcutrecht.nl.
